# Enhancing Typhoid Fever Diagnosis Based on Clinical Data Using a Lightweight Machine Learning Metamodel

**DOI:** 10.3390/diagnostics15050562

**Published:** 2025-02-26

**Authors:** Fariha Ahmed Nishat, M. F. Mridha, Istiak Mahmud, Meshal Alfarhood, Mejdl Safran, Dunren Che

**Affiliations:** 1 Dhaka National Medical College, Dhaka 1100, Bangladesh; farihaahmednishat@gmail.com; 2 Department of Computer Science, American International University-Bangladesh, Dhaka 1229, Bangladesh; 3 Department of Electrical and Electronic Engineering, Ahsanullah University of Science and Technology, Dhaka 1208, Bangladesh; istiakmahmud.eee@gmail.com; 4 Department of Computer Science, College of Computer and Information Sciences, King Saud University, P.O. Box 51178, Riyadh 11543, Saudi Arabia; malf@ksu.edu.sa; 5 Department of Electrical Engineering and Computer Science, Texas A & M University-Kingsville, Kingsville, TX 78363, USA; dunren.che@tamuk.edu

**Keywords:** typhoid fever diagnosis, machine learning metamodel, clinical data analysis, ensemble learning, non-invasive diagnostics, predictive modeling

## Abstract

**Background:** Typhoid fever remains a significant public health challenge, especially in developing countries where diagnostic resources are limited. Accurate and timely diagnosis is crucial for effective treatment and disease containment. Traditional diagnostic methods, while effective, can be time-consuming and resource-intensive. This study aims to develop a lightweight machine learning-based diagnostic tool for the early and efficient detection of typhoid fever using clinical data. **Methods:** A custom dataset comprising 14 clinical and demographic parameters—including age, gender, headache, muscle pain, nausea, diarrhea, cough, fever range (°F), hemoglobin (g/dL), platelet count, urine culture bacteria, calcium (mg/dL), and potassium (mg/dL)—was analyzed. A machine learning metamodel, integrating Support Vector Machine (SVM), Gaussian Naive Bayes (GNB), and Decision Tree classifiers with a Light Gradient Boosting Machine (LGBM), was trained and evaluated using k-fold cross-validation. Performance was assessed using precision, recall, F1-score, and area under the receiver operating characteristic curve (AUC). **Results:** The proposed metamodel demonstrated superior diagnostic performance, achieving a precision of 99%, recall of 100%, and an AUC of 1.00. It outperformed traditional diagnostic methods and other standalone machine learning algorithms, offering high accuracy and generalizability. **Conclusions:** The lightweight machine learning metamodel provides a cost-effective, non-invasive, and rapid diagnostic alternative for typhoid fever, particularly suited for resource-limited settings. Its reliance on accessible clinical parameters ensures practical applicability and scalability, potentially improving patient outcomes and aiding in disease control. Future work will focus on broader validation and integration into clinical workflows to further enhance its utility.

## 1. Introduction

Typhoid fever is a life-threatening condition triggered by the bacterium *Salmonella Typhi* [1,2,3,4]. Despite significant advances in medical science, typhoid fever continues to pose a vital public health challenge [5,6,7], significantly in least developed countries where access to advanced diagnostic tools is limited [8]. Traditional diagnostic methods for typhoid fever, such as blood cultures [9,10] and the Widal test [11,12,13,14], although effective, are often time-consuming, costly, and require well-equipped laboratories and skilled personnel [11]. These limitations highlight the urgent need for alternative diagnostic approaches that are both efficient and accessible.

**Figure 1:**
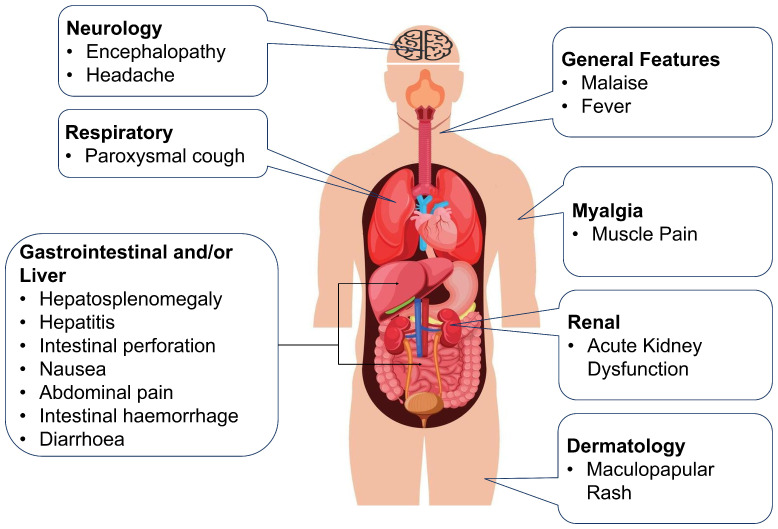
Systemic manifestations of typhoid fever: multiorgan impact and clinical features.

Figure 1 illustrates the systemic effects of typhoid fever, highlighting the critical impact on major organs and overall health [15]. Symptoms like fever, nausea, diarrhea, headache, muscle pain, and digestive distress often overlap with other illnesses, making it challenging to diagnose typhoid fever without extensive testing [16,17,18].

Given the limitations of current diagnostic approaches, there is a pressing need for a fast, efficient, and accurate predictive tool to forecast the presence of typhoid fever. Such findings would enable healthcare providers to establish early-stage treatment boldly and avoid further expansion of the disease.

Machine learning (ML) is one of the most potent tools in diagnosing health problems, offering the capability to assess complicated clinical data hurriedly and properly [19]. By utilizing big data records and high-level algorithms, machine learning models can detect sequences and interrelations that may not be instantly evident to healthcare providers [20,21]. This capability is particularly valuable in the context of typhoid fever, where earliest and reliable disease identification is important for efficient therapy and avoidance of outbreaks.

This research aims to develop a machine learning-based diagnostic tool for typhoid fever detection using clinical data. We worked on a custom dataset comprising various clinical parameters relevant to typhoid fever. The dataset includes attributes such as age, gender, headache, muscle pain, nausea, diarrhea, cough, fever range (°F), hemoglobin (g/dL), platelet count, urine culture bacteria, calcium (mg/dL), and potassium (mg/dL).

From this dataset, we hope to build a robust diagnostic tool by training a custom ML metamodel. We use a meta-ensemble technique to leverage different ML algorithms and generate a metamodel improving predictive power and generalizability. We utilize standard metrics to evaluate the model performance in a clinical setting.

Reducing the need for more expensive and invasive screening methods used currently, this research aims to yield a faster, more affordable, non-invasive diagnostic method for typhoid fever. In particular, the availability of such a tool would be a game changer for resource-limited settings, driving improved patient care, reduced disease transmission, and better utilization of our limited medical resources. This paper details the development and validation of our machine learning model, discussing its potential implications for the clinical management of typhoid fever. The primary contributions of this research are as follows:

- Design and implementation of a lightweight machine learning metamodel for the detection of typhoid fever.
- Validation of the proposed diagnostic tool through rigorous evaluation using standard performance metrics.
- Demonstration of a cost-effective and accessible diagnostic solution, particularly suited for resource-limited healthcare settings.
- Exploration of the broader implications of machine learning in enhancing clinical diagnostics and healthcare outcomes.

This paper is organized as follows: Section 2 provides a concise overview of the related works. Section 3 details the methodology employed in the study. The results are presented and analyzed in Section 4, while Section 5 delves into the discussion. Lastly, Section 6 concludes the paper, summarizing the key findings and insights.

## 2. Related Works

Typhoid fever has always been a critical public health issue, especially in low- and middle-income countries (LMICs), where diagnostic challenges are compounded by limited resources and inconsistent testing methods [22]. Research over the years has sought to address these challenges by improving prediction models, diagnostic techniques, and decision-support systems.

Table 1 illustrates related previous works by researchers. These works were selected through a systematic literature search using Google Scholar, PubMed, MDPI, IEEE Xplore, and Scopus. The search was conducted using relevant keywords such as “typhoid fever diagnosis”, “machine learning in typhoid”, “ensemble learning for typhoid disease prediction”, and “clinical data-based diagnosis of typhoid”. Inclusion criteria consisted of peer-reviewed articles, conference proceedings, and high-impact journal papers published in the last 10 years, focusing on machine learning applications in typhoid diagnosis and related infectious diseases. Studies lacking sufficient methodological details or with small, non-representative datasets were excluded to ensure relevance and reliability.

Shashvat et al. [23] investigated time-series models, including ARIMA, exponential smoothing, and support vector regression, to predict typhoid case trends in northern India. The study found exponential smoothing as the most effective method with the lowest prediction errors. However, its reliance on historical surveillance data limits its utility for real-time diagnostic applications, particularly in resource-constrained settings where timely diagnosis is critical. Zhang et al. [24] compared traditional SARIMA models with neural network-based approaches for forecasting typhoid fever incidence in China. While the radial basis function neural network outperformed others, the study’s focus on aggregate incidence forecasting neglects individual-level diagnostics and early detection, which are crucial for reducing morbidity and mortality.

Muche et al. [14] conducted a cross-sectional study assessing typhoid prevalence and associated factors among febrile patients in Ethiopia. Although the study identified key risk factors such as occupation and BMI, it relied on the Widal test, which has limited diagnostic accuracy and often leads to delayed or incorrect treatments, highlighting the need for more reliable diagnostic tools.

Antillón et al. [25] developed a meta-regression model to estimate typhoid burden across low- and middle-income countries. The study emphasized economic and environmental predictors but did not address diagnostic delays, which remain a significant barrier to effective treatment and disease control. Oguntimilehin et al. [26] and Obamiyi et al. [27] developed machine learning-based systems for diagnosing typhoid fever. While these studies demonstrated high classification accuracies, their reliance on small, localized datasets limits generalizability. Additionally, they do not leverage ensemble methods to enhance predictive performance, which could improve robustness and applicability across diverse settings.

Bhuiyan et al. [29] explored machine learning and deep learning techniques for predicting typhoid fever before clinical testing. The XGBoost classifier achieved the highest accuracy. However, the study’s focus on large feature sets and advanced algorithms may hinder implementation in resource-limited settings where computational resources are constrained. Samuel et al. [28] proposed a fuzzy logic-driven web-based decision support system for diagnosing typhoid. While the system offers flexibility, its dependence on fuzzy logic alone may limit accuracy compared to hybrid machine learning approaches that could better handle complex, multivariate clinical data.

Tran et al. [32] combined machine learning with high-content imaging to predict ciprofloxacin susceptibility in Salmonella Typhimurium. While innovative, this approach targets antimicrobial resistance rather than diagnostic accuracy for typhoid fever. Moreover, its imaging requirements and reliance on sophisticated equipment limit scalability in low-resource environments.

To address these gaps and serve these purpose simultaneously, this paper present a simple implementation metamodel-based diagnostic tool for typhoid fever. Including clinical parameters such as hemoglobin concentration, platelet count, and electrolyte levels enhances its clinical relevance and practicality. The model demonstrates high performance metrics (99% precision, AUC: 1.00) and could be deployed in resource-limited settings. And it has binding robustness and generalizability by a custom dataset as well as the efficient incorporation of advanced ensemble techniques in this work.

## 3. Methodology and Materials

**Figure 2:**
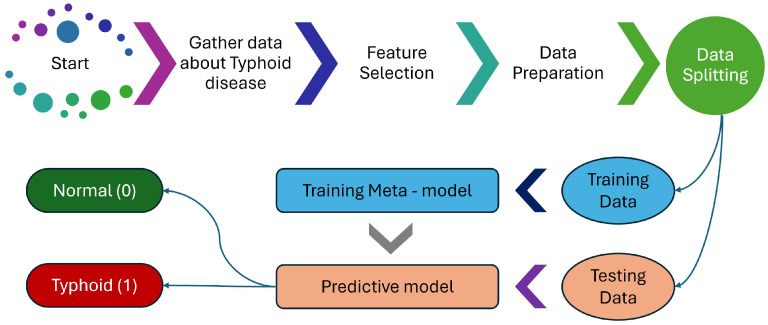
Proposed system’s flow diagram.

This study’s methodology focuses on creating and assessing an ML metamodel designed to predict typhoid using clinical test data. Figure 2 outlines a comprehensive process for developing a predictive model to analyze typhoid disease, with an additional focus on identifying typhoid disease. The process begins with gathering extensive data related to typhoid disease from various sources, ensuring a robust dataset that captures all relevant aspects of the disease. Feature selection was performed using a combination of domain expertise and statistical analysis to retain the most relevant clinical parameters while ensuring computational efficiency. Once the features were selected, the data underwent preparation, involving cleaning to remove inconsistencies, normalizing to ensure uniformity, and formatting for analysis. Proper data preparation is essential for ensuring the accuracy and reliability of the predictive model.

Once the data were processed, we split them into training and testing parts. During this process, the training data helps to build the metamodel based on the learning of patterns and relationships among the data, and acts as an intermediate step to a better-performing final model. Using the trained metamodel, a predictive model was developed for analyzing new data and predicting combinations based on learned patterns. To evaluate the model performance on unseen data, we made use of testing data. To conclude, the predictive model classifies new cases as either normal (no typhoid disease) or having typhoid disease based on the specified input variables, which can be used to easily identify an outcome. This algorithm assures an accurate predictive modeling process of typhoid disease from data collection, data cleaning, data pre-processing, to final classification.

### 3.1. Dataset and Attributes

The dataset used in this study was manually collected from patients who were either majorly or minorly affected by typhoid, as well as individuals who exhibited similar symptoms but were not diagnosed with typhoid based on medical reports [33]. These records were obtained from multiple medical clinics and hospitals across Bangladesh, ensuring a diverse and representative dataset for robust model evaluation. Diagnosis was determined through blood culture or the Widal test, specifically detecting the presence of *Salmonella Typhi*.

Initially, this dataset was gathered with the objective of applying AI-based analysis for improved disease detection. However, it has since been made publicly available to support further research and advancements in the field.

**Figure 3:**
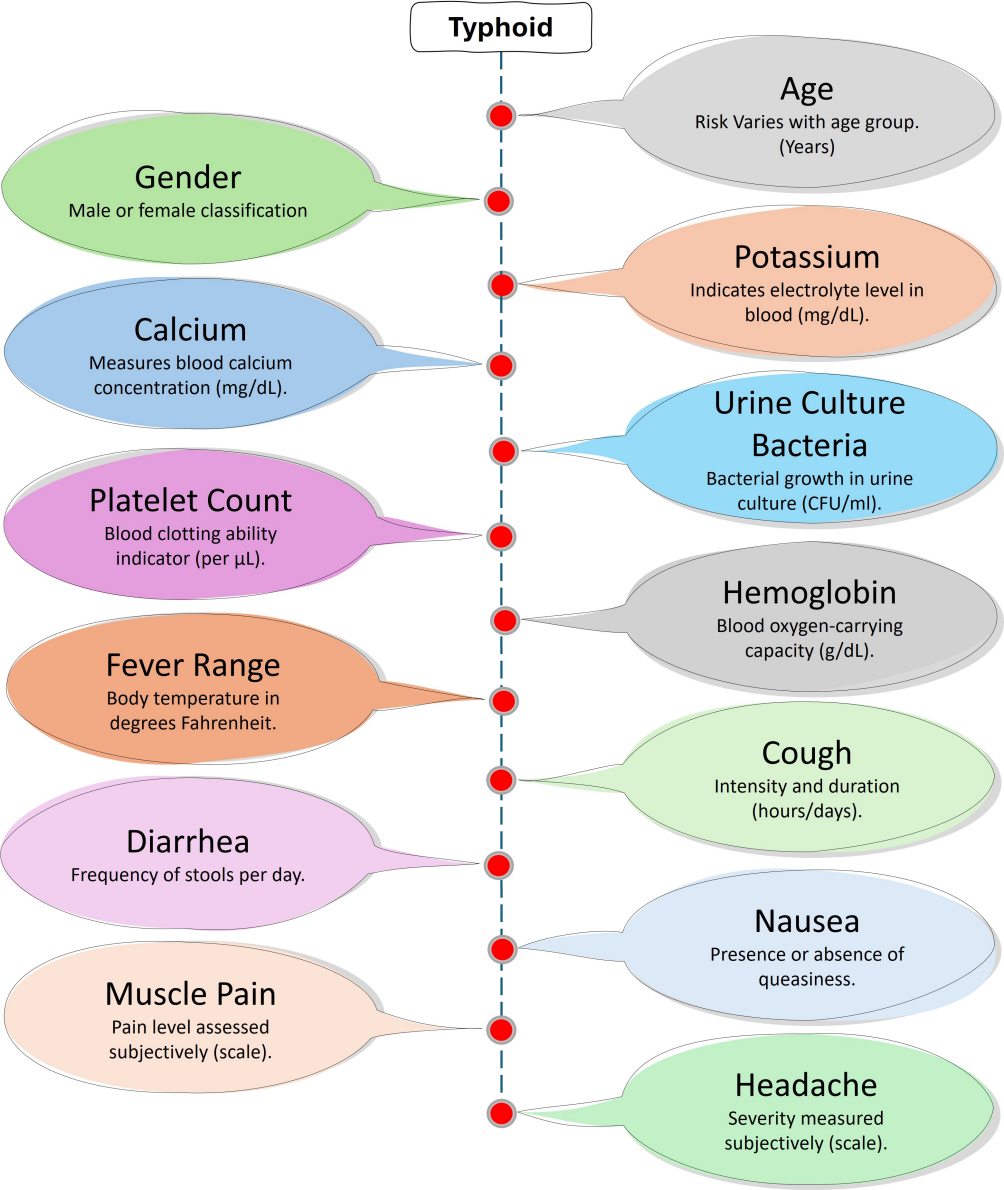
Key factors and symptoms associated with typhoid fever.

The provided dataset [33] contains comprehensive medical and demographic information for 659 individuals, aimed at analyzing the presence and impact of typhoid. This dataset is structured with 14 columns, each representing a specific attribute related to the individuals’ health and demographics, as shown in Figure 3. The attributes include age, gender, symptoms (like headache, muscle pain, nausea, diarrhea, and cough), fever range in degrees Fahrenheit, hemoglobin levels in grams per deciliter, platelet count, urine culture bacteria results, calcium levels in milligrams per deciliter, potassium levels in milligrams per deciliter, and a binary indicator for the presence of typhoid.

The “Age” attribute contains integer values representing the age of the individuals, providing a demographic perspective. The “Gender” column is a binary variable, distinguishing between male and female individuals. Symptoms like headache, muscle pain, nausea, diarrhea, and cough are also binary variables, recorded with “yes” or “no” responses to indicate the presence or absence of these symptoms. These features are critical for understanding the clinical presentation of typhoid in different individuals.

The dataset [33] also includes continuous medical data such as the “Fever Range (deg F)”, which captures the fever temperature range in degrees Fahrenheit. Hemoglobin levels, listed under “Hemoglobin (g/dL)”, and platelet counts are essential hematological parameters that offer insights into the individuals’ blood health. The “Urine Culture Bacteria” column identifies the type of bacteria found in urine cultures, providing important microbiological data with entries like “normal”, “Escherichia coli”, and “Klebsiella pneumoniae”.

In addition, the dataset [33] contains electrolyte levels, with “Calcium (mg/dL)” and “Potassium (mg/dL)” columns, respectively, indicating the concentration of these essential minerals in the bloodstream. This is important for evaluating the metabolic health of the individuals. Finally, the “Typhoid” column is a binary indicator of whether a person has typhoid or not, where “0” indicates that a person does not have typhoid and where “1” indicates that he/she has it. Such an occurrence is binary; the health profiles of the affected individuals are compared to those of unaffected individuals.

The first few lines of the dataset to show that there is a lot of diversity and range in the data collected. Example: 24-year-old male with typhoid, temp range = 101.8 °F, hemoglobin = 13.7 g/dL, platelet count = 272,673. It helps in making a diagnosis and excluding differential diagnoses, for example, the values for a 37-year-old female patient without typhoid with a body temperature of 100.4 °F, hemoglobin of 13.2 g/dL, and a platelets count of 257,447. These differences illustrate how typhoid may affect other health indicators differently across individuals.

This dataset [33] is a comprehensive snapshot of health indicators, symptoms, and demographic data to understand the presence and effect of typhoid. It tracks vital clinical data such as hemoglobin levels, platelet counts, fever temperatures, and electrolyte levels along with scores of symptoms and bacterial cultures. This set of detailed data is useful for discovering patterns and correlations between the presence of typhoid and multiple health parameters, which can further aid medical analysis and research.

### 3.2. Data Analysis and Preprocessing

Preprocessing involved checking for missing values, but no null values were found in the dataset. Preprocessing involved converting categorical features into numerical values using Label Encoding [34]. Numerical features were then standardized using StandardScaler to ensure a uniform scale. These preprocessing steps ensured that the data was appropriately structured for machine learning algorithms.

**Figure 4:**
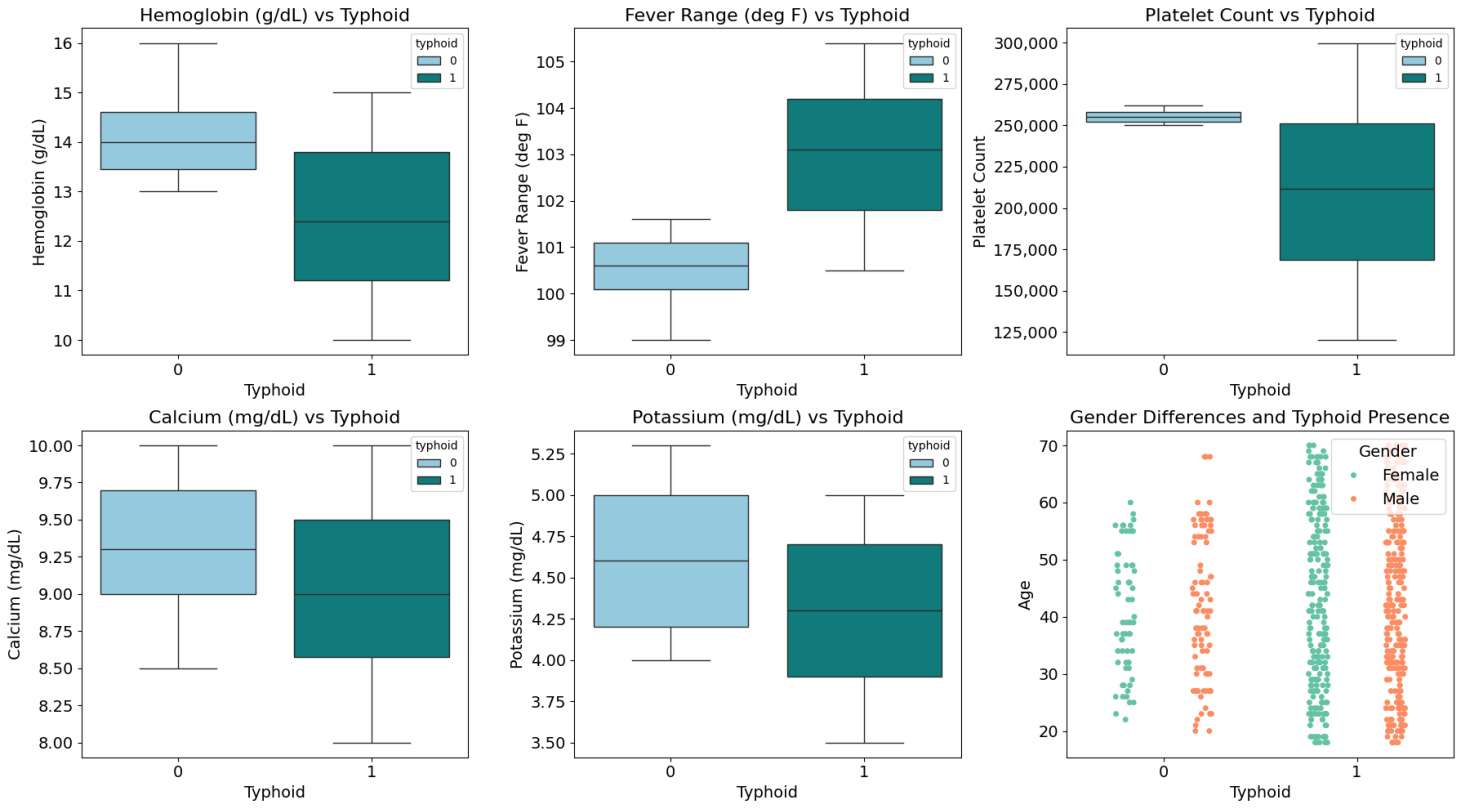
Clinical parameters and gender differences in typhoid diagnosis.

Figure 4 illustrates the variation in clinical parameters between typhoid patients and other patients without typhoid fever, and is valuable for interpreting diagnostic data. The top-left graph shows a significant drop in hemoglobin levels in typhoid patients compared to non-typhoid patients. This could imply that typhoid fever has equal chance with reduced hemoglobin concentrations, which could relate to either the systemic inflammatory response found in patients with typhoid fever or anemia (both paths frequently appearing in typhoid patients). This reduction in hemoglobin levels could be a valuable indicator for distinguishing typhoid from other febrile illnesses.

The top-middle graph in Figure 4 reveals that fever range is notably higher in typhoid patients, with temperatures predominantly exceeding 102 °F. In contrast, non-typhoid cases tend to exhibit fever temperatures below this threshold, reinforcing the significance of elevated and sustained fever as a hallmark symptom of typhoid fever. This distinction in temperature ranges can serve as a key diagnostic parameter when combined with other clinical indicators. The top-right graph highlights platelet count differences, showing that typhoid patients experience significantly lower counts with a broader distribution. Platelet counts in typhoid cases drop as low as 125,000, while non-typhoid individuals exhibit consistently higher values. This could reflect the impact of typhoid on bone marrow suppression or systemic inflammation, both of which are known to influence platelet production and survival. These findings emphasize the need to consider platelet levels in the diagnostic process.

Moving to the lower row in Figure 4, the bottom-left graph shows that calcium levels are slightly reduced in typhoid patients, typically concentrated below 9.5 mg/dL. Non-typhoid individuals, on the other hand, tend to have higher calcium values on average. This observation may point to underlying metabolic or nutritional disturbances associated with typhoid fever, which could provide additional diagnostic clues. Similarly, the bottom-middle graph reveals that potassium levels are generally lower in typhoid patients, clustering in the range of 3.5–4.5 mg/dL. In contrast, non-typhoid cases exhibit a wider and higher range of potassium levels. This variation might be associated with dehydration or gastrointestinal losses, which are common in typhoid fever, and could further support differential diagnosis.

Finally, the bottom-right scatter plot in Figure 4 visualizes the distribution of age and gender in relation to typhoid presence. The dataset consists of 659 patients with 14 clinical and demographic attributes. Among them, 500 are diagnosed with typhoid, while 159 are not. The dataset includes 354 male and 305 female patients. Typhoid cases are distributed across all age groups, with a slightly higher prevalence in males, possibly due to behavioral or occupational factors. The data highlights key clinical indicators such as hemoglobin reduction, fever elevation, platelet suppression, and electrolyte imbalances, reinforcing their role in enhancing early and accurate diagnosis, especially in resource-limited settings.

**Figure 5:**
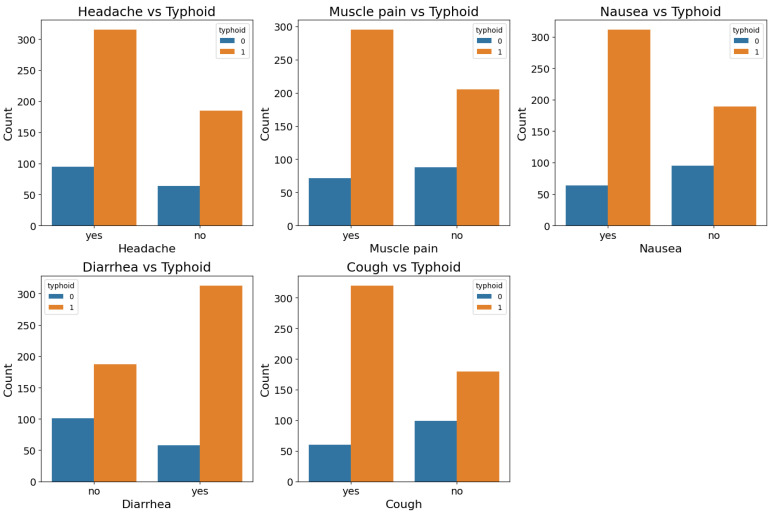
Comparison of symptom prevalence (headache, muscle pain, nausea, diarrhea, and cough) between typhoid and non-typhoid cases.

Figure 5 provides a detailed overview of the relationship between common binary symptoms—headache, muscle pain, nausea, diarrhea, and cough—and the presence of typhoid fever. Across all graphs, individuals diagnosed with typhoid (typhoid = 1) exhibit a significantly higher count of positive symptoms compared to those without typhoid (typhoid = 0). These trends highlight the diagnostic significance of these symptoms in differentiating typhoid cases from other febrile illnesses. In the headache plot (top-left), the majority of typhoid-positive patients report experiencing headaches, whereas this symptom is far less common in non-typhoid individuals. This pattern underscores headache as one of the frequent clinical manifestations associated with typhoid fever. A similar trend is observed in the muscle pain graph (top-middle), where a much larger proportion of typhoid-positive individuals report muscle pain compared to non-typhoid cases. This observation may reflect the systemic inflammatory response typically seen in typhoid fever.

The nausea graph (top-right in Figure 5) also follows a similar pattern where typhoid-positive individuals have more counts of nausea than non typhoid individuals. This adds nausea as a major symptom to the clinical picture of typhoid fever. These trends highlight the need for evaluating combinations of these symptoms that may help in the identification of typhoid suspects. The difference is even more stark in the diarrhea graph (bottom-left). Diarrhea is reported by a large proportion of typhoid patients, while only a small percentage of non-typhoid patients have the same symptom. This pronounced contrast emphasizes the cardinality of diarrhea in the symptomatology of typhoid fever, which frequently aids in differentiating it from other diseases that present similar symptoms.

Finally, the cough graph (bottom-right in Figure 5) illustrates that typhoid-positive individuals frequently report having a cough, whereas the non-typhoid group shows significantly fewer instances of this symptom. This trend indicates that while cough may not be as universal as diarrhea or headache, it still plays a contribution in the constellation of symptoms indicative of typhoid fever. These visualizations collectively demonstrate that symptoms like headache, muscle pain, nausea, diarrhea, and cough are not only more prevalent in individuals with typhoid fever but also serve as significant clinical indicators. Recognizing these symptom patterns is crucial for early detection and diagnosis, particularly in resource-constrained settings where using advanced diagnostic tools may be limited. This analysis underscores the value of incorporating these features into diagnostic models to improve the accuracy and efficiency of typhoid fever detection.

**Figure 6:**
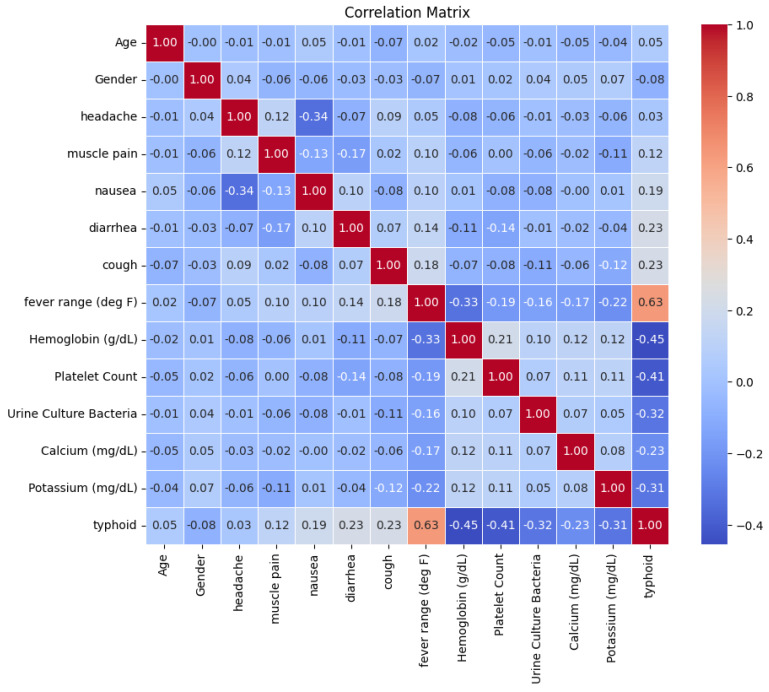
Correlation matrix of clinical and laboratory parameters in typhoid cases.

Figure 6 provides a detailed overview of the relationships between various clinical and laboratory parameters and typhoid fever. Among the features, “fever range (deg F)” exhibits the highest positive correlation (0.63) with typhoid, indicating that elevated fever is a strong indicator of the disease. Additionally, nausea (0.19), diarrhea (0.23), and cough (0.23) show moderate positive correlations, reinforcing their common presence in typhoid-positive patients.

In contrast, several laboratory parameters in Figure 6 demonstrate negative correlations with typhoid. Hemoglobin (−0.45), platelet count (−0.41), potassium (−0.31), and urine culture bacteria (−0.32) are negatively associated with the disease, suggesting that lower values of these features are commonly observed in typhoid patients due to its hematological and metabolic impact.

Although age, gender, and calcium display weak correlations in Figure 6 with typhoid, they were retained in the model due to their contextual and predictive significance. Age can influence disease severity and immune response, while gender-based physiological differences may affect symptom manifestation. Calcium levels, though weakly correlated, could interact with other variables in nonlinear ways that correlation metrics alone do not capture. Machine learning models leverage feature interactions, and removing variables solely based on correlation could lead to loss of valuable information and reduced model generalization.

The diagonal values of 1.0 in the correlation matrix in Figure 6 represent self-correlations, ensuring the validity of the dataset structure. Overall, the matrix highlights key clinical indicators—fever range, hemoglobin, platelet count, and diarrhea—that significantly contribute to typhoid diagnosis while underscoring the importance of retaining all relevant features for a more robust predictive model.

### 3.3. Proposed Machine Learning Metamodel

**Figure 7:**
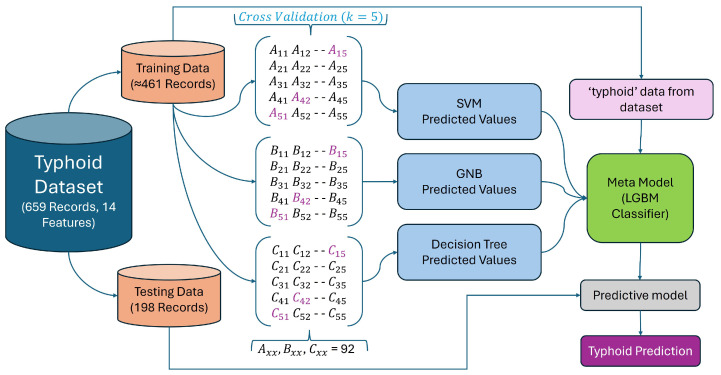
Ensemble learning framework for typhoid prediction using multiple classifiers and metamodel integration.

The complete working diagram of the proposed machine learning metamodel is illustrated in Figure 7. The typhoid dataset used in this study consists of 659 records and 14 features, covering various clinical and demographic parameters relevant to typhoid fever diagnosis. The dataset was split into 70% training data (≈461 records) and 30% testing data (198 records) to ensure that the model learns from a substantial portion of the data while still retaining enough samples for independent testing and evaluation.

During the training phase, the training data (≈461 records) were used to train three different base classifiers: SVM [35,36], Gaussian Naive Bayes (GNB) [37,38], and Decision Tree (DT) [39,40,41]. Each classifier independently analyzed the data and produced predicted values based on its learned decision boundaries. To improve generalization and reduce overfitting, 5-fold cross-validation was applied during training. In this process, the training data were split into five subsets, where four subsets were used for training and one for validation in each iteration. This ensured that every data point contributed to both training and validation at different stages, leading to a more robust model.

Once the base classifiers generated their predictions, the next step was metamodel integration. Instead of relying on the outputs of SVM, GNB, and Decision Tree separately, their predicted values were fed into a LightGBM (LGBM) classifier, which served as the metamodel. Additionally, the actual ‘typhoid’ labels from the dataset were provided as input to the metamodel during training, allowing it to learn patterns from the combined outputs of the base classifiers. This stacked ensemble approach enhances predictive performance by leveraging the strengths of multiple models and reducing individual weaknesses.

After training, the predictive model was applied to the testing data (198 records) to evaluate its performance. The trained metamodel processed the unseen data and generated final typhoid prediction results. The ensemble learning framework, combining multiple classifiers and refining their predictions through a metamodel, ensures improved accuracy and reliability in typhoid fever diagnosis.

The selection of Support Vector Machine (SVM), Gaussian Naive Bayes (GNB), and Decision Tree (DT) as base models in our metamodel was based on a balance between accuracy, interpretability, and computational efficiency. SVM is effective for high-dimensional feature spaces, GNB provides a fast probabilistic approach for categorical clinical data, and Decision Tree enhances interpretability while capturing complex patterns. Other classifiers were initially tested but were not included due to lower predictive accuracy or computational inefficiency. Some models required significantly more training time without meaningful accuracy improvements, while others lacked the interpretability necessary for clinical decision making. The combination of these three classifiers ensures a robust and generalizable diagnostic model by leveraging their complementary strengths.

### 3.4. Different Machine Learning Algorithms

The proposed machine learning model utilizes a metamodel approach incorporating four different models. SVM, Gaussian Naïve Bayes (GNB), and Decision Tree serve as the base models, while LightGBM functions as the final classifier, forming the core of our metamodel architecture.

Additionally, to ensure a comprehensive performance comparison, we trained, tested, and validated our model against several other algorithms (Decision Tree [42], Gradient Boosting [43], K-Nearest Neighbors (KNN) [44], Linear Discriminant Analysis (LDA) [45], Logistic Regression [46], a blended Metamodel, Neural Network, or Multi-Layer Perceptron (MLP) [47], GNB [48], Support Vector Machine [49], and XGBoost [50]). The listed algorithms represent a mix of traditional and advanced ML techniques, each suited for specific tasks. Decision Trees work by dividing data into branches based on feature values, creating a tree-like model for classification or regression tasks. K-Nearest Neighbors (KNN) is a simple, non-parametric method that classifies a point by the majority vote of its nearest neighbors. GNB, a probabilistic model, is based on Bayes’ theorem and assumes independence among features while following a normal distribution, making it particularly efficient for handling categorical and continuous data in large datasets. LDA reduces dimensionality by transforming data into a lower-dimensional space, focusing on maximizing the separation between classes while minimizing variation within each class.

Algorithms such as Logistic Regression and SVMs form the bedrock of both binary and multi-class classification. SVMs can seem less intuitive than logistic regression, which uses a linear model to estimate the probabilities of classes that are transformed by the sigmoid function, as SVMs work by identifying the hyperplane that best separates the classes with the largest margin, which makes it more powerful in high-dimensional data. The ensemble methods like Gradient Boosting and XGBoost construct a strong learner from a set of weak learners, typically decision trees, which are learned sequentially. XGBoost is a gradient-boosted trees algorithm that generalizes it by adding regularization.

More complex methods like Neural Networks (MLP) and Metamodels are designed for intricate relationships in data. Neural networks, inspired by biological systems, use layers of interconnected neurons to model nonlinear relationships. These methods collectively form a comprehensive toolkit for solving diverse ML problems, from simple tasks to advanced predictive modeling.

## 4. Experimental Results

This section explains all the findings and output of the proposed ML models, as well as that of state-of-art ML models for comparison in terms of performance and validation.

### 4.1. Experimental Setup

The coding was performed on the Google Colab platform within a Python environment with version 3.9, utilizing the Scikit-learn library for data analysis [51].

The training process employs the Radial Basis Function (RBF) kernel for the SVM and the Gaussian kernel for Naïve Bayes (GaussianNB). The Decision Tree, Gradient Boosting, XGBoost, and LightGBM (LGBM) Classifiers are tree-based models that do not utilize explicit kernels. The MLP Classifier applies the Rectified Linear Unit (ReLU) activation function, which serves as an implicit kernel for feature transformation. The Linear Discriminant Analysis (LDA) model employs a linear transformation, effectively functioning as a linear kernel. Regarding hyperparameters, the MLPClassifier is configured with a maximum of 500 iterations for convergence. The XGBoost classifier is set with 50 estimators, with label encoding disabled and log loss as the evaluation metric. The Stacking Classifier is implemented with 5-fold cross-validation and uses the LightGBM (LGBM) Classifier as the final estimator.

### 4.2. Evaluation Metrics

Evaluation metrics help quantify how well a model performs on a given task and provide a basis for comparing different models, enabling comparisons between models and guiding improvements. We explain here the Confusion Matrix [52,53], Performance Matrix [54] and ROC-AUC [55].

#### 4.2.1. Confusion Matrix

**Figure 8:**
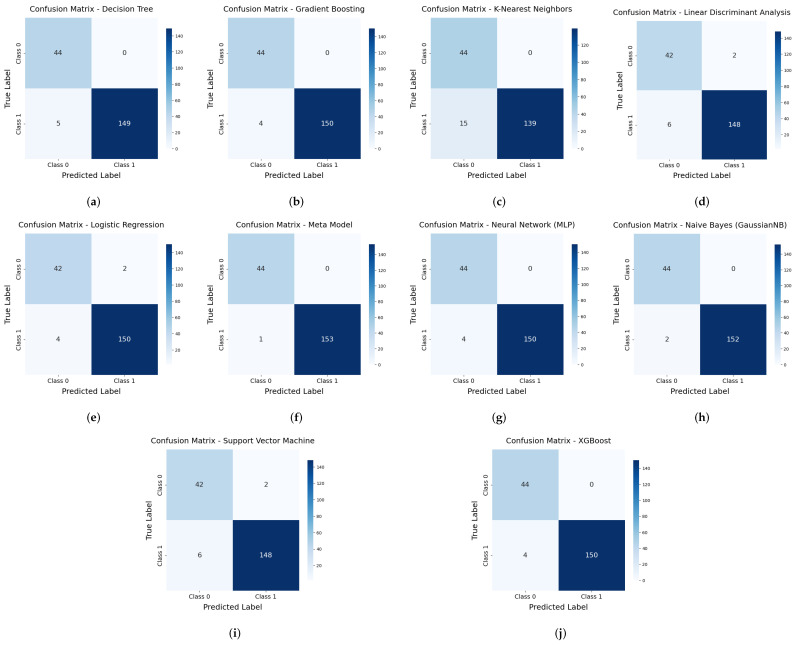
Confusion matrices for various machine learning models. (**a**) Decision Tree. (**b**) Gradient Boosting. (**c**) K-Nearest Neighbors. (**d**) LDA. (**e**) LogReg. (**f**) Metamodel. (**g**) MLP. (**h**) Naive Bayes. (**i**) SVM. (**j**) XGBoost.

To identify the most effective algorithm, several detection methods were applied to the dataset, and their performance was assessed based on accuracy and other statistical parameters. The evaluation involved comparing these methods using specific metrics. Figure 8 presents confusion matrices for different machine learning models.

Figure 8 shows the classification performance for two classes: Class 0 and Class 1. The diagonal elements indicate correctly classified instances, while the off-diagonal elements represent misclassifications. The Decision Tree model performs well, correctly identifying 44 instances of Class 0 and 149 of Class 1, with five misclassifications. Gradient Boosting improves on this, reducing the misclassification of Class 1 instances to four. Similarly, Logistic Regression and LDA exhibit comparable accuracy, with both models correctly classifying 42 instances of Class 0 while maintaining minimal misclassification rates. However, KNN shows weaker performance, with 15 Class 1 instances incorrectly classified as Class 0, making it the least effective among these models.

Deep learning and ensemble methods demonstrate higher accuracy and robustness. The Multi-Layer Perceptron (MLP) achieves perfect classification for Class 0 and four misclassifications of Class 1, suggesting that neural networks can effectively capture complex decision boundaries. Naïve Bayes (GaussianNB) performs very well, correctly classifying 152 instances of Class 1 while misclassifying two. SVM and XGBoost show strong performance, closely matching LDA but with slightly different misclassification distributions. These models generally provide improved classification accuracy over simpler approaches like KNN and Decision Trees.

A key takeaway from these results is that ensemble and hybrid models tend to perform best. Gradient Boosting and XGBoost refine predictions effectively, while the Metamodel, which combines multiple models, achieves the highest accuracy. By leveraging strengths from different models, the Metamodel correctly classifies all 44 Class 0 instances and 153 Class 1 instances, with the lowest misclassification rate among all approaches.

Overall, the Metamodel outperforms all other classifiers, demonstrating superior accuracy and robustness. This suggests that meta-learning techniques, which integrate multiple models, provide the best results for classification tasks, minimizing errors while maintaining high predictive reliability.

#### 4.2.2. Performance Matrix

Precision (P), recall (Re), F1-score (F1), and accuracy (Acc) are critical evaluation metrics in ML because they provide a comprehensive and multifaceted understanding of a model’s performance, particularly in classification tasks. Each of these metrics serves a distinct purpose by capturing specific aspects of the model’s ability to make correct predictions. Precision highlights the reliability of positive predictions by measuring how many of the predicted positive cases are actually correct. Recall focuses on the model’s sensitivity, or its ability to correctly identify all relevant positive instances. The F1 score balances these two metrics, offering a single, harmonic mean value that is particularly useful in scenarios with imbalanced datasets. Accuracy, on the other hand, gives an overall measure of correctness by determining the proportion of all predictions that are accurate. Together, these metrics are vital in assessing how well a model aligns with the problem requirements and in identifying trade-offs that might be necessary depending on the domain or application. They enable practitioners to make informed decisions about model suitability, ensuring robust and context-appropriate performance.

Accuracy is the ratio of correctly predicted observation to the total observation. It is good for balanced datasets but can be confusing for imbalanced datasets.

(1) $$\mathrm{Acc}=\frac{\mathrm{TP}+\mathrm{TN}}{\mathrm{TP}+\mathrm{TN}+\mathrm{FP}+\mathrm{FN}}$$

Precision or Positive Predictive Value looks at the number of true positive predictions compared to the total number of all positive predictions. It focuses on reducing false positives and is especially critical in cases where false alarms have severe implications.

(2) $$P=\frac{\mathrm{TP}}{\mathrm{TP}+\mathrm{FP}}$$

Recall measures how many actual positive cases are correctly identified by the model. It emphasizes minimizing false negatives and is crucial when missing positive cases is costly.

(3) Re=TPTP+FN

F1-score illustrates the harmonic mean of P and Re, offering a balanced metric that accounts for both. It is especially valuable for imbalanced datasets, as it incorporates the impact of false positives and false negatives.

(4) F1=2·P·ReP+Re

For Equations (1)–(4), the following terms are used: FP represents False Positives, TP denotes True Positives, P indicates Precision, FN refers to False Negatives, Re stands for Recall, TN represents True Negatives, and F1 corresponds to the F1-score.

Table 2 presents the classification performance of various machine learning models for typhoid detection, evaluated using precision, recall, and F1-score for both Class 0 (Non-Typhoid) and Class 1 (Typhoid cases). The Metamodel achieves the highest performance, with an F1-score of 0.9967 for Class 1, indicating its ability to correctly classify typhoid cases with minimal errors. Other models, such as Gradient Boosting, XGBoost, and Neural Networks (MLP), also perform exceptionally well, showing high precision and recall scores, making them reliable for detecting typhoid.

KNN shows weaker performance in Table 2, particularly for Class 0, with a low precision of 0.7458, meaning it frequently misclassifies non-typhoid cases as positive. In contrast, Gradient Boosting and XGBoost achieve F1-scores of 0.9868, demonstrating their strong classification capability. Naive Bayes (GaussianNB) and Neural Network (MLP) also perform well, both achieving F1-scores of 0.9935, making them effective choices for detecting typhoid cases with high accuracy.

Among all models in Table 2, the Metamodel emerges as the best performer, achieving the highest precision, recall, and F1-score, ensuring the most reliable and balanced classification for typhoid detection.

#### 4.2.3. Model Size, Root Mean Square Error (RMSE), and Accuracy

The Metamodel, a combination of multiple base learners, demonstrates exceptional efficiency with a model size of just 0.0259 MB (26 kB), making it a lightweight yet highly effective solution. In comparison, the Neural Network (MLP), which is typically considered one of the lighter deep learning models, has a significantly larger size of 0.0560 MB—more than twice the size of the Metamodel. Other models in the comparison, such as XGBoost (0.0447 MB) and K-Nearest Neighbors (0.1033 MB), also exhibit larger memory footprints, with Gradient Boosting reaching 0.1617 MB, the highest among them. While Neural Networks (NN) can be optimized for higher accuracy by adding hidden layers, this inevitably leads to an increase in model size, making them less suitable for real-time applications where memory and computational efficiency are critical. These findings highlight that the Metamodel provides an optimal balance between accuracy and resource consumption, making it a compelling choice over traditional deep learning approaches, particularly in environments with strict memory and computational constraints.

**Figure 9:**
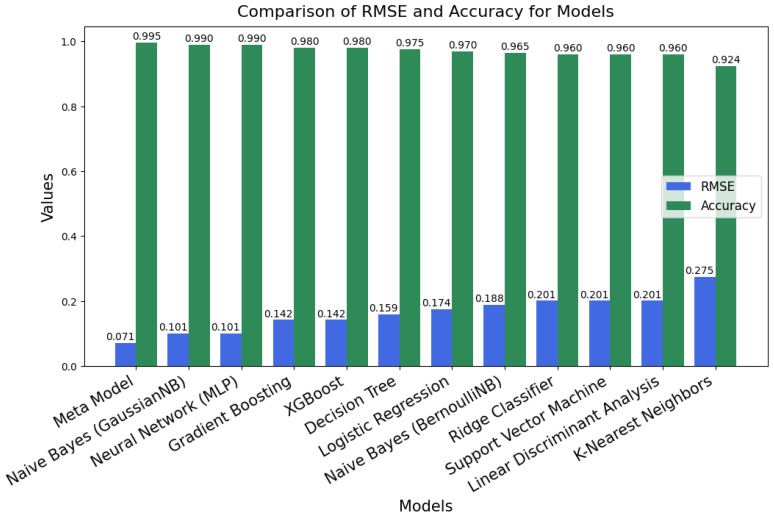
Performance comparison of ML models based on RMSE and Accuracy metrics.

The bar chart in Figure 9 compares the Root Mean Square Error (RMSE) and Accuracy for various ML models. The green bars represent accuracy values, while the blue bars represent RMSE values, where higher accuracy and lower RMSE indicate better model performance. Among the models, the Metamodel clearly stands out as the best performer, achieving the highest accuracy of 0.995 and the lowest RMSE of 0.071, showcasing its ability to make highly precise predictions with minimal error.

Several other models in Figure 9, including the Naive Bayes and MLP (Neural Network), also perform strongly, achieving accuracies close to 0.990 with RMSE values around 0.101. These models demonstrate a good balance of accuracy and error minimization, performing reliably across the evaluation metrics.

In contrast, the KNN model shows the weakest performance in Figure 9, with the lowest accuracy at 0.924 and the highest RMSE at 0.275. This indicates its relative difficulty in generalizing well for the given task compared to other models. Overall, the Metamodel emerges as the best performer, with its near-perfect accuracy and minimal RMSE, making it the most precise and reliable model among all the compared algorithms.

#### 4.2.4. ROC-AUC

**Figure 10:**
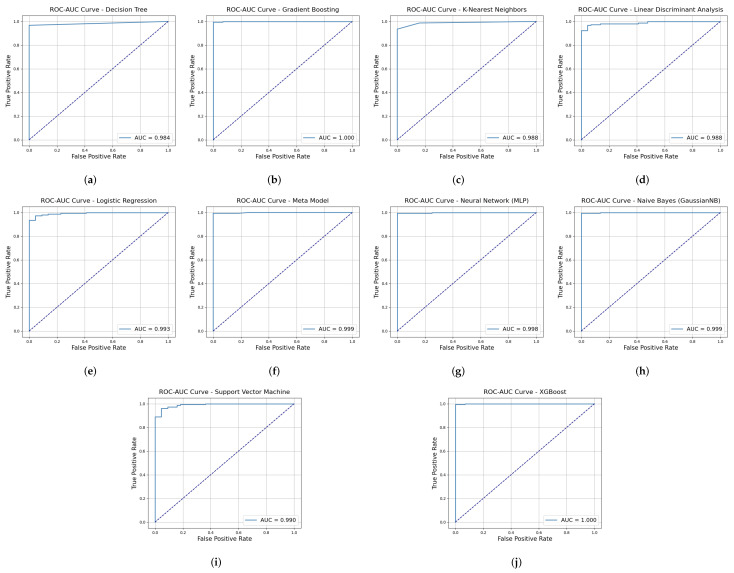
ROC-AUC curves for various machine learning models. (**a**) Decision Tree. (**b**) Gradient Boosting. (**c**) K-Nearest Neighbors. (**d**) LDA. (**e**) Logistic Regression. (**f**) Meta Model. (**g**) Neural Network (MLP). (**h**) Naive Bayes. (**i**) Support Vector Machine. (**j**) XGBoost.

Figure 10 shows that the Metamodel stands out as the best performer in this analysis, achieving a flawless AUC of 1.00. This indicates its unparalleled ability to classify the data perfectly, with the ROC curve reaching the ideal top-left corner. By combining predictions from multiple models, the Metamodel leverages the strengths of each to deliver exceptional classification performance.

Analyzing other models in Figure 10, Decision Tree, Gradient Boosting, Neural Network (MLP), Naive Bayes, and XGBoost achieved AUC scores of 0.99 without significant deviations, which is close to the Meta Model’s performance. However, KNN, LDA, Logistic Regression, and SVM showed slightly lower performance compared to the other models. While these models demonstrate near-perfect classification performance, their ROC curves exhibit slight deviations compared to the Metamodel, suggesting occasional misclassifications or sensitivity to specific data characteristics.

## 5. Discussion

This research illustrates the prospect of a lightweight ML metamodel as a transformative tool for the diagnosis of typhoid fever, particularly in resource-limited settings. By integrating multiple ML algorithms into an ensemble approach, the metamodel achieves exceptional diagnostic performance, with a precision of 99%, recall of 100%, and an AUC of 1.00. These results significantly surpass the early diagnostic limitations of traditional methods like the Widal test, which suffer from high false-positive rates, variability, and resource-intensive implementation.

The proposed work stands out as the most comprehensive and effective approach to typhoid fever detection, addressing key gaps and limitations in previous researches as illustrates in Table 1. Unlike earlier studies that relied fewer comparative models, or lacked statistical and machine learning integration, our approach employs ensemble learning (SVM, GNB, Decision Tree, and LGBM) to enhance diagnostic accuracy. Achieving 99% precision, 100% recall, and an AUC of 1.00, it significantly outperforms traditional and previous methods. Furthermore, it resolves issues related to generalizability, external validation, computational efficiency, and feature optimization—critical challenges identified in prior works. By leveraging clinical data for robust machine learning-based diagnostics, our model ensures a more reliable, efficient, and scalable solution for real-world medical applications.

The proposed metamodel capitalizes on accessible clinical parameters such as platelet counts, fever range, and common symptoms, making it a practical, non-invasive diagnostic alternative. Its ability to generalize across the dataset while minimizing computational complexity enhances its applicability in real-world scenarios, particularly in areas where advanced diagnostic infrastructure is unavailable.

The use of proposed metamodel enhances diagnostic accuracy and robustness but introduces interpretability challenges due to its ensemble structure. Unlike standalone models, the integration of SVM, GNB, Decision Tree, and LGBM adds abstraction, making it harder to trace individual feature contributions. However, we mitigate this by analyzing feature importance, ensuring transparency in decision making. Deep learning and highly nonlinear models were excluded due to their computational complexity and limited practicality for real-time applications. While the meta-classifier reduces interpretability, the selected ensemble approach maintains a balance between accuracy, efficiency, and explainability. The inclusion of interpretable base models, such as decision trees, helps balance accuracy and interpretability. Future work may incorporate explainability techniques like SHapley Additive exPlanations (SHAP) or Local Interpretable Model Agnostic Explanation (LIME) to further enhance model transparency while maintaining high predictive performance.

Deep learning models, such as deep neural networks (DNNs) and convolutional neural networks (CNNs), typically require large-scale datasets and high computational resources, making them less suitable for real-time clinical applications in resource-constrained environments. Instead, we employed the Multi-Layer Perceptron (MLP) Classifier, a lightweight neural network model that effectively balances predictive performance and computational efficiency, ensuring real-time feasibility for typhoid fever diagnosis. However, our proposed metamodel outperforms all standalone models, including MLP, achieving superior accuracy, recall, and AUC while maintaining computational efficiency, making it the optimal choice for practical deployment.

A comparative analysis with other state-of-the-art ML methodologies demonstrates the metamodel’s superiority, achieving the best results across all validation metrics. While previous studies using neural networks and fuzzy logic systems show promise, their complexity and data dependency limit scalability. In contrast, the lightweight nature and modular architecture of the metamodel allow for ease of deployment on low-resource systems, ensuring both scalability and cost-effectiveness.

From a clinical standpoint, the high sensitivity of the metamodel ensures minimal false negatives, which is crucial in mitigating the risks associated with delayed typhoid fever diagnosis. Furthermore, its reliance on readily available clinical data underscores its potential for rapid implementation in diverse healthcare settings, including rural and underserved regions. Yet, the results are also emphasize the need to validate across larger and more diverse datasets to ensure broad applicability and consistency of approach.

Despite these achievements, some limitations remain. The geographic scope of the dataset necessitates validation in other regions to account for population-specific variations in clinical parameters. Additionally, the incorporation of temporal data, co-existing medical conditions, and environmental factors could further enhance the model’s robustness and accuracy. Future work should also explore the integration of this metamodel into real-time diagnostic workflows and assess its usability from the perspective of healthcare practitioners.

The dataset used in this study is imbalanced, with approximately 24.13% of the records representing normal (non-typhoid) cases and 75.87% being typhoid-positive cases. Class imbalance can lead to misleading accuracy metrics, as a model biased toward the majority class may achieve high accuracy by predicting most cases as typhoid-positive while failing to correctly classify normal cases. To ensure a fair performance assessment, we incorporated additional evaluation metrics such as precision, recall, F1-score, and ROC-AUC, which provide a more comprehensive view of the model’s effectiveness. High recall is particularly crucial in medical diagnosis, as missing actual typhoid cases (false negatives) could have serious health consequences. Our results show consistently high recall and F1-score values, indicating that the model effectively balances sensitivity and specificity despite the dataset imbalance. While techniques such as oversampling or class-weight adjustments could be explored to further address this issue, our model’s strong performance across multiple evaluation metrics suggests that it generalizes well without the need for additional balancing methods.

In summary, this study provides a strong foundation for the development and deployment of lightweight ML-based diagnostic tools for typhoid fever. The proposed metamodel stands out as a promising solution for addressing the diagnostic challenges of typhoid fever in resource-constrained environments, with the potential to significantly improve healthcare delivery and patient outcomes.

## 6. Conclusions

This research introduces a lightweight ML metamodel that provides a robust, time-effective, and non-invasive diagnostic alternative for typhoid fever. By leveraging accessible clinical parameters and advanced ensemble learning techniques, the metamodel achieves near-perfect diagnostic accuracy, demonstrating its potential to significantly enhance healthcare outcomes, particularly in resource-constrained settings.

The findings underscore the metamodel’s utility in overcoming the limitations of traditional diagnostic tools, offering faster, more reliable, and scalable solutions for typhoid fever detection. Its modular design and reliance on non-invasive data make it adaptable for diverse healthcare environments, from rural clinics to urban hospitals. Moreover, the high sensitivity and specificity of the model ensure timely and accurate diagnosis, enabling better patient management and disease containment.

Future work should focus on validating the model across larger and more diverse datasets, integrating additional clinical and environmental parameters, and assessing real-world deployment in clinical workflows. By addressing these areas, the metamodel could further solidify its role as a transformative tool for typhoid fever diagnosis, contributing to improved global health outcomes. 

## Figures and Tables

**Table 1:** Summary of research papers on typhoid fever, including their purposes, key findings, identified gaps, and the use of machine learning.

Previous Researches	Purpose	Key Findings	Gap Identified	Use of Machine Learning
Ajibola et al. [8]	Review of typhoid fever diagnostics in endemic countries	Widal test is unreliable; newer PCR methods show promise.	Lack of sensitive and specific diagnostics in low-resource settings.	No, diagnostic focus
Abayneh et al. [13]	Assess prevalence of typhoid, typhus, and malaria in Southwest Ethiopia	11.3% for *Salmonella Typhi*, 10.2% for *Rickettsia*, and 11.2% for malaria among febrile patients.	Reliance on serological tests with reduced specificity.	No
Shashva et al. [23]	Evaluate effectiveness of different time series models	Exponential smoothing model outperforms others	used Fewer models for comparison	Yes, support vector machine regression
Zhang et al. [24]	Compare forecasting models for typhoid fever incidence	RBFNN outperforms other models	Computationally Complex for Real-Time Implementation	Yes, neural network models
Antillon et al. [25]	Assess baseline burden of typhoid in LMICs	17.8 million typhoid cases annually, highest in Central Africa and parts of Asia	Limited incidence data, need for better surveillance	No, statistical modeling and meta-regression
Oguntimilehin et al. [26]	Evaluation of rapid diagnostic tests for typhoid	Many rapid tests have low sensitivity and specificity	Cost and applicability challenges in endemic areas	No, focus on diagnostic tools
Obamiyi et al. [27]	Develop a mobile application for typhoid fever diagnosis	Multilayer Perceptron achieved high accuracy in classifying typhoid fever cases.	Limited to data collected from a single hospital.	Yes, Multilayer Perceptron (MLP)-based model implemented in a mobile application.
Samuel et al. [28]	Develop a web-based decision support system using fuzzy logic for diagnosis.	Fuzzy logic system achieved 94% efficiency in diagnosing typhoid fever based on signs, symptoms, and laboratory test results.	Need for improved clinical decision systems in rural areas.	Yes, fuzzy logic-based medical diagnosis system.
Bhuiyan et al. [29]	Predict typhoid fever before clinical trials using machine learning models	XGBoost classifier achieved 97.87% accuracy; ANN and other models were also effective.	Scope of accuracy improvement and computationally intensive	Yes, multiple algorithms, including XGBoost and ANN, applied.
Odion et al. [30]	To develop a web-based diagnostic system for typhoid and malaria detection.	Typhoid Binary Classifier (TBC) achieved 96.1% accuracy	The study only focuses on XGBoost and does not compare its performance with other ensemble models.	Yes, XGBoost
Apanisile et al. [31]	To develop an AI-driven extended diagnostic system (EDS) for malaria and typhoid fever detection	The system analyzes symptoms and predicts the likelihood of malaria or typhoid using probability-based inference.	Limited Machine Learning Model Comparisons	Yes, Naïve Bayes Classifier

**Table 2:** Comparison of model performances with reporting features for typhoid detection.

Model	Class	Precision	Recall	F1-Score
Logistic Regression	0	0.9130	0.9545	0.9333
	1	0.9868	0.9740	0.9804
Decision Tree	0	0.8980	1.0000	0.9462
	1	1.0000	0.9675	0.9835
Gradient Boosting	0	0.9167	1.0000	0.9565
	1	1.0000	0.9740	0.9868
Support Vector Machine	0	0.8750	0.9545	0.9130
	1	0.9867	0.9610	0.9737
K-Nearest Neighbors	0	0.7458	1.0000	0.8544
	1	1.0000	0.9026	0.9488
Naive Bayes (GaussianNB)	0	0.9565	1.0000	0.9778
	1	1.0000	0.9870	0.9935
Neural Network (MLP)	0	0.9565	1.0000	0.9778
	1	1.0000	0.9870	0.9935
LDA	0	0.8750	0.9545	0.9130
	1	0.9867	0.9610	0.9737
XGBoost	0	0.9167	1.0000	0.9565
	1	1.0000	0.9740	0.9868
Metamodel	0	0.9901	1.0000	0.9888
	1	0.9935	1.0000	0.9967

## Data Availability

The original contributions presented in this study are included in the article. Further inquiries can be directed to the corresponding author.

## Abbreviations

The following abbreviations are used in this manuscript:

MLP	Multi-Layer Perceptron
LDA	Linear Discriminant Analysis
SVM	Support Vector Machine
GNB	Gaussian Naive Bayes
LGBM	Light Gradient Boosting Machine
KNN	K-Nearest Neighbors
AUC	Area Under the Curve
ROC	Receiver Operating Characteristic
RMSE	Root Mean Square Error
PCR	Polymerase Chain Reaction
LMICs	Low- and Middle-Income Countries
DT	Decision Tree
GBoost	Gradient Boosting
XGBoost	Extreme Gradient Boosting
LogReg	Logistic Regression
DNN	Deep Neural Networks
FN	False Negative
FP	False Positive
TP	True Positive
P	Precision
Re	Recall
TN	True Negative
F1	F1-Score
Kb	Kilobyte
MB	Megabyte
LIME	Local Interpretable Model Agnostic Explanation
SHAP	SHapley Additive exPlanations
